# Reply: Breast cancer and induced abortions in China

**DOI:** 10.1038/sj.bjc.6601855

**Published:** 2004-05-26

**Authors:** D B Thomas, R M Ray

**Affiliations:** 1 Fred Hutchinson Cancer Research Center, Program in Epidemiology, 1100 Fairview Avenue North, PO Box 19024, M4-13847, Seattle, WA 98109-1024, USA

**Sir**,

In their letter with regard to our paper on induced abortions and breast cancer, Brind and Chinchilli essentially suggest that residual confounding by age at first birth and parity may have caused us to underestimate the odds ratio (OR) for breast cancer in relation to induced abortion. We disagree. In paragraph 2 of their letter, they suggest that women in China who did not have an induced abortion would be more likely to be nulliparous and to have had their children later in life than women who had an abortion, that the women unexposed to abortions were therefore at higher risk of breast cancer than those with an abortion, and that the true OR in relation to induced abortion was thus underestimated. This is not correct. Few women in our study cohort were nulliparous and, as stated in our paper, the results were virtually unchanged when the analyses were restricted to gravid or parous women. Because of the one child per family policy in China, which became operational in the early 1980s, older women in our study tended to have larger numbers of children than younger women, and to have begun child bearing at an earlier age. Because of this, after controlling for age, the number of children was not a confounder, and age at first birth was only a weak confounder. During the time period covered by our study, abortions were almost always performed to limit family size. The decision to have an abortion would thus have been made after the birth of ones first child. Therefore, age at first birth would not necessarily be earlier for women with an abortion than for women of the same age without an abortion, as Brind and Chinchilli contend.

Brind and Chinchilli point out that our crude OR for breast cancer in relation to induced abortion is 0.93, and our OR adjusted for age and age at first birth is 1.06. In the next paragraph, they suggest that confounding by parity and age at first birth would somehow not be fully controlled for by adjustment because of the high prevalence of induced abortion (51%) in our study population, and therefore that the OR of 1.06 should actually be higher. We fail to understand how the prevalence of the exposure could directly influence the confounding effect of other factors. If one were to (hypothetically) conduct a randomised trial of abortion and breast cancer, the most efficient design would be to assign 50% of the women to an abortion group and 50% to a control group. Confounding would be controlled for by the randomisation. In our study, confounding was essentially controlled for by stratification on the potentially confounding variables of concern. With a prevalence of exposure to abortions of 51%, we have the optimal power to detect a true association and to control for confounding. If the prevalence of abortions in the population were closer to 0%, it would have been more difficult to control for confounding by stratification because of smaller numbers in the exposed group.

The contention in the sixth paragraph of the letter that the lower relative risk in the study by Sanderson than in our study is due to more residual confounding as a result of a higher prevalence of abortions in the Sanderson study is unwarranted. There are many reasons for differences in results between studies. In fact, the results of the Sanderson study and ours are very close (ORs of 1.0 and 1.06, respectively), and provide consistent evidence that induced abortions probably do not cause breast cancer.

In the case–control study nested within our cohort, controls were matched to cases on exact year of birth, and the potentially confounding effects of parity and age at first birth were controlled for in the statistical analysis. Brind and Chinchilli suggest that we should have also matched on these two factors. We disagree with their contention that this would have given a more accurate estimate of the OR. Our sample size was large enough to allow us to control tightly for these two variables. Tight control is as good a method for controlling for confounding as equally tight matching.

In their last paragraph, they note that we failed to cite a paper by Howe *et al*. This is one of over 50 studies, and our paper was not meant to be a comprehensive review of the literature. We are aware of six large cohort studies in addition to our own, all of which had results showing no increase in risk of breast cancer in relation to induced abortion. The contention by Brind and Chinchilli that ‘the overwhelming majority of published studies indicate a positive association between induced…abortion and breast cancer incidence’ is simply false. The reference that they give for this statement is a paper by Brind! A workshop, attended by over 100 expert participants, on Early Reproductive Events and Breast Cancer was sponsored by the US National Cancer Institute, and held in February of this year. A major conclusion from that workshop was that induced abortions do not increase the risk of breast cancer. We believe that this issue has been satisfactorily laid to rest.

